# Natural products as potential drug treatments for acute promyelocytic leukemia

**DOI:** 10.1186/s13020-024-00928-8

**Published:** 2024-04-03

**Authors:** Jiaxin Chen, Zuoqi Ding

**Affiliations:** 1 School of International Pharmaceutical Business, China Pharmaceutical University, Nanjing, China; 2 Editorial Board of Chinese Journal of Natural Medicines, Nanjing, China

**Keywords:** Acute promyelocytic leukemia, Natural products, Traditional Chinese medicine, ATO, RIF, Sesquiterpene lactones

## Abstract

Acute promyelocytic leukemia (APL), which was once considered one of the deadliest types of leukemia, has become a curable malignancy since the introduction of all-trans retinoic acid (ATRA) and arsenic trioxide (ATO) as clinical treatments. ATO, which has become the first-line therapeutic agent for APL, is derived from the natural mineral product arsenic, exemplifying an important role of natural products in the treatment of APL. Many other natural products, ranging from small-molecule compounds to herbal extracts, have also demonstrated great potential for the treatment and adjuvant therapy of APL. In this review, we summarize the natural products and representative components that have demonstrated biological activity for the treatment of APL. We also discuss future directions in better exploring their medicinal value, which may provide a reference for subsequent new drug development and combination therapy programs.

## Introduction

### The onset and treatment of acute promyelocytic leukemia

According to the latest World Cancer Report released by the International Agency for Research on Cancer of the World Health Organization (WHO), in 2020, there were 474,519 new cases of leukemia globally, accounting for 2.5% of all cancer cases, and 311,594 new leukemia deaths, accounting for 3.1% of all cancer deaths [1]. Acute promyelocytic leukemia (APL) is a subtype of leukemia that was first defined as a specific form of acute myeloid leukemia (AML) in 1957 [2]. APL accounts for 10–15% of AML cases [3]. APL is characterized by a severe tendency to hemorrhage in the early stage. The incidence of thrombosis in APL is higher than that of other types of leukemia, and the treatment process is susceptible to hemorrhage and hemothrombosis, which may lead to the death of patients [4, 5]. The bone marrow of patients with APL is dominated by abnormal granulocytic proliferation of promyelocytes, which accounts for more than 30% of non-erythroid nucleated cells. The pathogenesis of APL is related to chromosomal translocation. More than 98% of patients with APL have *PML*-*RARα* gene fusion, which is due to t (15;17) gene translocation, resulting in the fusion of promyelocytic leukemia (*PML*) on chromosome 15 with the gene encoding retinoic acid receptor-α (*RARα*) on chromosome 17, producing the fusion protein *PML-RARα*, which prevents cancer cell apoptosis [6, 7].

**Fig. 1:**
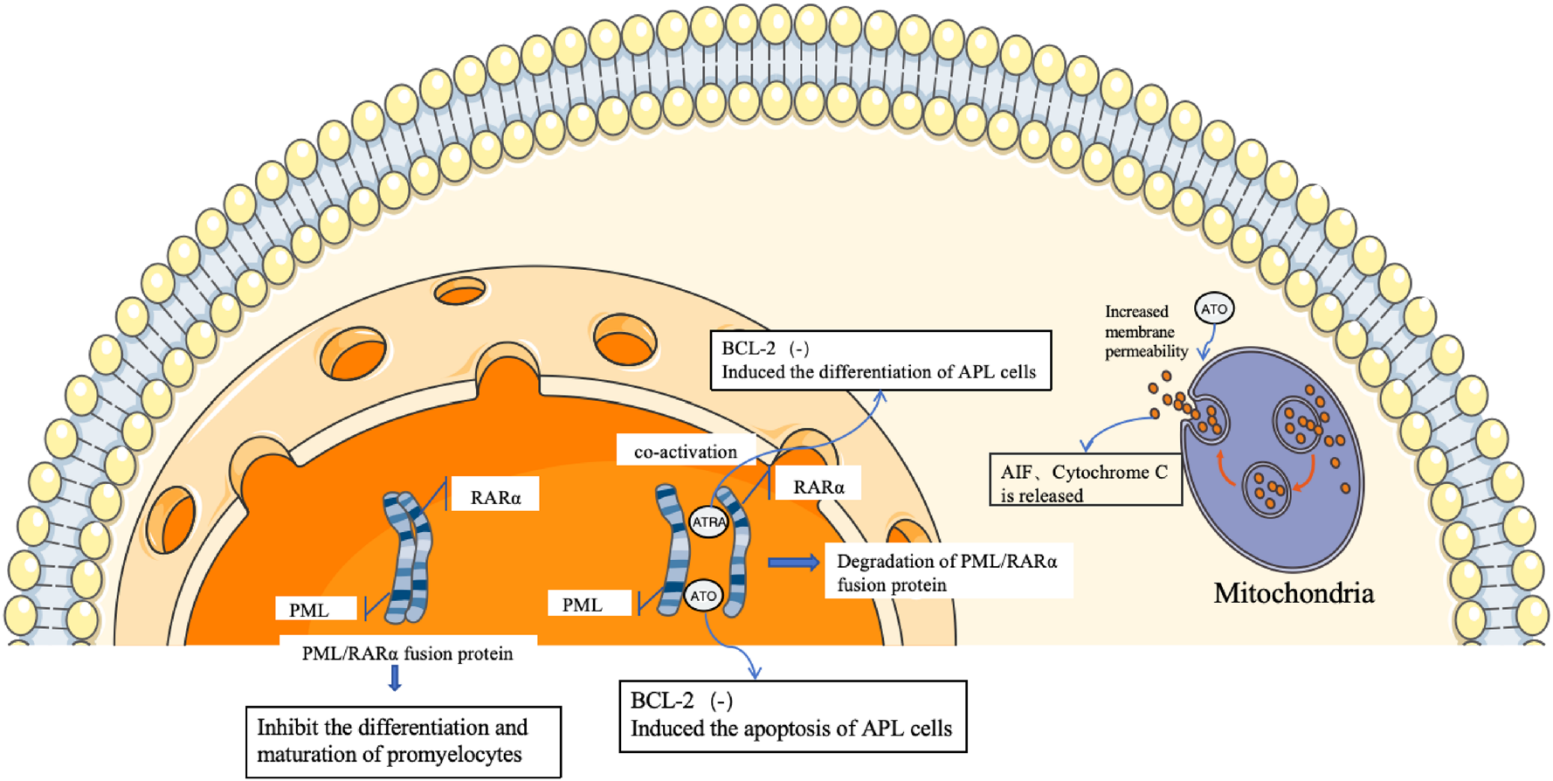
Mechanisms of ATRA and ATO in the treatment of APL. The *PML-RARα* fusion protein inhibits the differentiation and maturation of promyelocytes through dominant-negative inhibition. ATRA degrades *PML-RARα*, and ATO increases mitochondrial pore permeability, resulting in the release of apoptosis-inducing factor (AIF) and cytochrome C to the outside of the mitochondria, inducing apoptosis

APL was once considered one of the deadliest types of leukemia due to its rapid disease progression and high early hemorrhagic mortality. Before the introduction of anthracyclines in the clinic, the survival of patients with APL was 3–16 weeks. The introduction of anthracyclines made the complete remission (CR) rate of APL comparable to that of other AML subtypes [8]. In the 1980s, both all-trans retinoic acid (ATRA) and arsenic trioxide (ATO) were first applied to the clinical treatment of APL by Chinese scholars, which led to epochal advances in the treatment and prognosis of this disease [9, 10], their mechanisms for treating APL is shown in Fig. 1. International authoritative guidelines, including the 2023 edition of the *National Comprehensive Cancer Network Clinical Guidelines for Acute Myeloid Leukemia* [11], the 2018 edition of *the Chinese Guidelines for the Diagnosis and Treatment of Acute Promyelocytic Leukemia* [3], and the *Management of Acute Promyelocytic Leukemia by the Expert Panel of the European LeukemiaNet* [12], recommend ATRA and ATO as first-line treatment options for APL. Due to the standardized clinical use of ATRA and arsenic agents, the treatment of APL does not rely on hematopoietic stem cell transplantation, and APL has become one of the most curable malignant diseases [13].

### APL treatment by traditional prescription

Before the introduction of standardized treatment, leukemia treatment in China often relied on the use of traditional Chinese medicine (TCM). Many early clinical observations have demonstrated that TCM has a beneficial impact on the treatment of leukemia. Ailing No.1 is an injection containing arsenolite and light powder. It is the predecessor of ATO injections, which are currently widely used as the first-line treatment of APL. Since the 1970s and 1980s, there have been clinical observations on the treatment of APL by the combination of Ailing No.1 and TCM dialectical treatment, achieving an overall effective rate of 78% and a CR rate of 59% [14]. Among the TCM prescriptions used were Lianhua Decoction, Xiangsha Six Gentlemen Decoction, Longdan Xiegan Decoction, Qinggu Powder, Angong Niuhuang Pill, Zixue Pill, etc., which can help regulate the immune system and prevent infections in clinical practice. Qinghuang powder, as a typical example, is a TCM prescription recorded in the *Effective Formulae Handed Down for Generations* (Yuan Dynasty) and *Wonderful Well-Tried Recipes* (Qing Dynasty), and its main ingredients are *indigo naturalis* and realgar. In the 1980s, Zhou et al. began using Qinghuang powder to treat acute non-lymphoblastic leukemia. They successfully cured two patients with M3, providing evidence of its beneficial impact on APL [15]. In the 1990s, clinical observations were conducted on the treatment of leukemia using a combination of Qinggu Powder and herbal medicines like *Hedyotis diffusa*, and the overall effective rate of this prescription in treating leukemia reached 84.6% [16]. Xiaochengqi Decoction from the *Prescriptions for Universal Relief* (Ming dynasty) plus *Hedyotis diffusa* and antibiotics can treat severe infections complicated by acute leukemia [17]. Since TCM prescriptions typically consist of natural medicines, the aforementioned clinical records demonstrate the positive impact of natural medicines in treating leukemia. With advancements in science and technology, numerous natural products found in traditional prescriptions have been proven to possess biological activity for APL treatment. Detailed discussions on some of the active ingredients are presented below. Table 1 displays several TCM prescriptions utilized for leukemia treatment.

**Table 1:** Traditional Chinese medicine prescriptions and their possible active ingredient in the treatment of leukemia

Traditional Chinese medicine prescriptions	Possible active ingredients in the treatment of leukemia	Source of natural medicine
Lianhua Decoction	Oleanolic acid	*Forsythia suspensa* (Thunb.) Vahl
Xiangsha Six Gentlemen Decoction	Dehydrocostus lactone	*Aucklandia lappa* Decne
Longdan Xiegan Decoction	Oleanolic acid, crocin and crocetin	*Akebia quinata*(Thunb.)Decne., *Gardenia jasminoides* Ellis
Qinggu Powder	Artesunate	*Artemisia annua* L
Angong Niuhuang Pill	As_2_S_2_, oleanolic acid	Realgar, *Akebia quinata*(Thunb.)Decne
Zixue Pill	Dehydrocostus lactone	*Aucklandia lappa* Decne
Qinghuang Powder	As_2_S_2_, crocin and crocetin	Realgar, *Gardenia jasminoides* Ellis
Xiaochengqi Decoction	Honokiol	*Magnolia grandiflora*

### APL treatment by natural products

Natural medicines play an important role in the clinical treatment of APL in China. Arsenic and realgar, which are mineral medicines based on arsenic compounds, have been used as treatments for thousands of years. Nowadays, these two medicines are still utilized in the era of modern medicine. Arsenic acid, the aqueous solvent of the main ingredient of arsenic, is made into an injection and often combined with ATRA for the treatment of APL. There is no cross-resistance between ATRA and ATO, and patients with APL who are refractory to ATRA and conventional chemotherapeutic agents are still able to undergo treatment with ATO with good responses [18]. The oral arsenic, Realgar-*Indigo Naturalis* Formula (RIF), which is composed entirely of TCM with realgar as the main ingredient, is also widely used in clinical practice. In the *Chinese Guideline for Diagnosis and Treatment of Childhood Acute Promyelocytic Leukemia* published in the *Chinese Journal of Applied Clinical Pediatrics* in 2022, RIF is recommended as the first choice of arsenic agent [19].

Natural medicines play a unique role in the treatment of malignant tumors. In addition to RIF, which is compatible with wisdom, early clinical treatment of APL is often accompanied by TCM evidence-based treatment. This approach has achieved good therapeutic results, with few side effects and good therapeutic efficacy, supporting a great clinical application value [14]. The 2014 edition of the *Chinese Guidelines for the Diagnosis and Treatment of Acute Promyelocytic Leukemia* has also introduced homoharringtonine (HHT) [20]. HHT is an anti-leukemia drug for clinical application, which was first recommended in China. It is derived from *Cephalotaxus* and has anti-tumor activity [21]. In addition, as the therapeutic scope of ATO is often limited due to its cardiotoxicity and many natural medicines play a protective role in the treatment of ATO-induced cardiac side effects, natural medicines have broad prospects as cardioprotective agents against ATO-induced cardiac side effects [22].

Most natural products used in the treatment of cancer have multiple targets with multiple biological properties. Therefore, they can treat a wide range of diseases, making them preferable over single-target drugs [23]. Among the anti-tumor medicines used in clinical practice with high efficacy, 60% are obtained from natural products [24]. Identifying active ingredients from natural products and developing them into drugs for clinical use fully promotes the utilization of natural resources. In recent years, due to the continuous development of science and technology, the treatment of tumors has not been limited to traditional chemotherapy. Cell therapy, gene therapy, and other emerging treatment modalities have gradually increased the curative rate of malignant tumors, but the high cost of cell therapy has become a major concern. In light of this, the inexpensiveness, safety, and effectiveness of natural products make them a viable option for the treatment of malignant tumors, such as leukemia. Focusing on APL, this review attempts to summarize the natural medicines with therapeutic potential for APL, which is expected to serve as an updated reference for future drug development.

## Natural medicines currently used for APL treatment

### Arsenic and realgar derived from minerals

Mineral medicines are non-renewable resources that encompass natural minerals, processed minerals, and fossilized animal bones. China has a long history of using mineral medicines, and the Soil Department and the Gold and Stone Department in the *Compendium of Materia Medica* have recorded the use of various types of mineral medicine, including realgar, orpiment, and arsenic, which contain arsenic as their main active ingredient. In the periodic table, the element As (arsenic) is located below P (phosphorous), belonging to the VA group. As such, arsenic compounds are similar to phosphorus in many ways. For example, the common oxidation state is As (III) and As (V), the state in nature is generally As_2_O_3_, and it can form As (OH)_3_ or its corresponding arsenite (AsO_2_^-^) in water. Of these, As (V) is less toxic, and its biological activity is based primarily on phosphate substitution [25]. At present, both arsenic and realgar are used as first-line treatments for APL, especially ATO injection, the efficacy of which is widely recognized worldwide.

#### The “poison”—ATO

In China, the use of arsenic as a treatment for leukemia dates back to the 1970s [26]. Dr. Tingdong Zhang of the First Affiliated Hospital of Harbin Medical University was inspired by a rural doctor’s prescription for treating skin cancer and used arsenic, light powder, and bufalin to configure a prescription for the small-range treatment of cancer. The patients achieved different degrees of improvement, and it was later confirmed that the active ingredient in the prescription was arsenic. Later, it was found that the efficacy of arsenic alone was comparable to the original formula, and, ATO, the main component of arsenic, was therefore directly used to design ATO injections [27]. Arsenic was previously considered as a highly toxic drug, and its use in clinical practice was limited to oral or topical application. China was the first country to develop it into an intravenous solution for the treatment of leukemia [6]. Nowadays, the treatment of APL is standardized, and ATRA combined with ATO could achieve an APL cure rate of more than 90% [28]. A prospective randomized clinical trial of ATRA-ATO/ATRA-chemotherapeutic agents (NCT01987297) showed that disease-free survival (DFS) was achieved in 96.1% of patients in the ATO group compared with 92.6% in the non-ATO group, and the 7-year relapse rate was significantly lower in the ATO group. During consolidation follow-up, grade 3–4 hematologic toxicity was significantly reduced in the ATO group. This shows that ATRA-ATO is not inferior to ATRA-chemotherapy in consolidating replacement chemotherapy and chemotherapy reduction [29]. According to the latest recommendations of the Expert Panel of European LeukemiaNet, the use of ATO in children with APL not only reduces exposure to high cumulative doses of anthracyclines, thereby reducing some long-term side effects, but it also improves outcomes in populations with a higher prevalence of high-risk disease [12]. Additional clinical trials have shown that ATRA-ATO has significantly greater and durable anti-leukemic efficacy than ATRA-chemotherapeutic agents in low-/intermediate-risk APL, and the advantages of ATRA-ATO will likely be demonstrated over time [30].

Arsenic, as a traditional medicine, is known for its toxicity. Arsenic is one of the 10 chemicals listed by the WHO as being a major public health concern. Inorganic arsenic has been recognized as a carcinogen and is the most significant chemical contaminant in drinking water worldwide [31]. In terms of whether the use of ATO will aggravate the burden in patients with APL, pharmacokinetic studies in Japan have demonstrated that ATO is metabolized when administered intravenously to patients with APL and that its methylated metabolites are rapidly eliminated from the bloodstream and excreted into the urine upon dosing completion, indicating no measurable accumulation of ATO in the bloodstream [32]. Hepatotoxicity, particularly in terms of increased liver enzymes, is often reported with the use of ATO. However, this type of hepatotoxicity is largely reversible and can be successfully managed by reducing or temporarily discontinuing ATO, and fatal liver failure has rarely been reported [33].

Although intravenous administration has become an effective treatment route for ATO, there is an emerging trend toward the use of oral ATO. Oral ATO does not put patients at risk of developing venous thrombosis, which can lead to a much better quality of life and convenience for patients, as well as reducing the burden on healthcare facilities compared with intravenous administration [34]. Ventricular arrhythmias occur in approximately 30% of patients during intravenous administration of ATO. When arsenic is administered orally, peak plasma arsenic is decreased and cardiotoxicity is relatively low [35]. Oral arsenic has obvious safety advantages, and if reliable clinical trials prove that it is not less effective than intravenous ATO, oral arsenic will be a great advancement in the treatment of APL.

#### Realgar as another arsenic compound

**Fig. 2:**
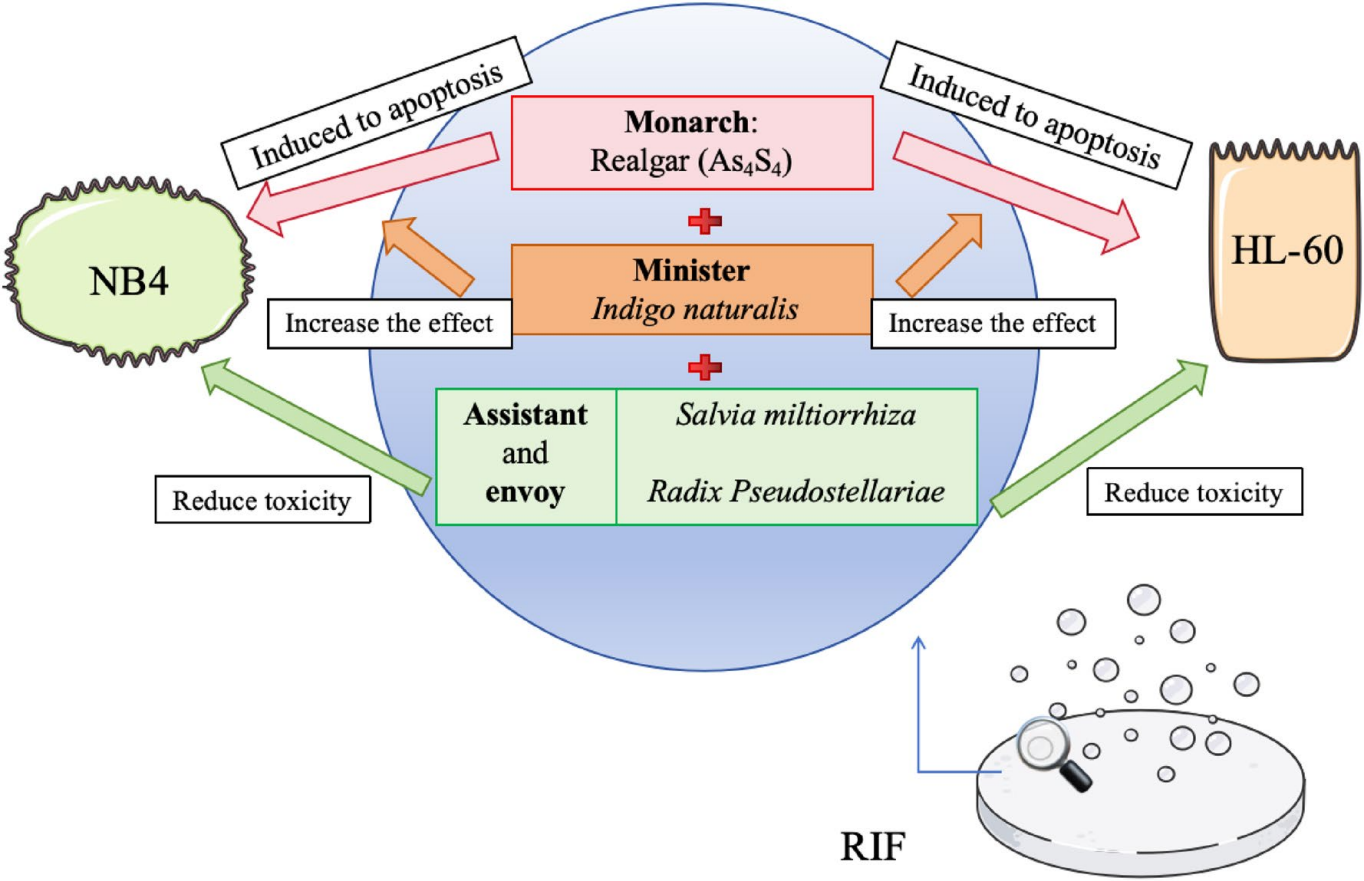
RIF follows the important formula of “Monarch, Minister, Assistant, and Envoy”. Realgar induces apoptosis in NB4 cells and HL-60 cells, indigo naturalis increases the efficacy of realgar, and the combination of *Salvia miltiorrhiza* and radix *Pseudostellariae* reduces arsenic toxicity

Realgar is a mineral medicine that is commonly used in TCM, and its main component is As_2_S_2_ with a small amount of As_2_O_3_ and other metal salts. In 1980, Shilin Huang, a professor at the 210th Hospital of the People’s Liberation Army, formulated a pure TCM complex formula, namely Fufang Huangdai Tablet (RIF). RIF follows the important formula of “Monarch, Minister, Assistant and Envoy,” as shown in Fig. 2, with realgar as the “Monarch,” *indigo naturalis* as the “Minister,” and *Salvia miltiorrhiza* and radix *Pseudostellariae* as the “Assistant and Envoy” [36]. Realgar is the main component in the formula, and its main active ingredient is As_2_S_2_, which induces apoptosis in NB4 cells and HL-60 cells, and its effect is enhanced with time and concentration within certain ranges [37, 38]. *Indigo naturalis* increases the efficacy of realgar, and the combination of *Salvia miltiorrhiza* and *Radix Pseudostellariae* can reduce arsenic toxicity [39]. There is also clinical evidence showing that RIF used in combination with ATO is less cardiotoxic than intravenous ATO alone, and oral RIF also reduces the incidence of infection compared with ATO [40]. A clinical trial examining the efficacy of intravenous ATO versus oral RIF demonstrated that RIF-ATRA was not inferior to ATO-ATRA for the treatment of patients with non-high-risk APL. This study suggests that a completely oral, chemotherapy-free model may be an alternative to standard intravenous therapy for patients with non-high-risk APL [41].

RIF was the first commercially available oral arsenic agent approved in China, and it is commercially available in mainland China, with no license for use in other regions. The treatment of APL in the majority of patients is moving toward being completely oral without the use of chemotherapy. Oral RIF with ATRA will ultimately make treatment safer, reduce the economic burden, and increase the accessibility to more patients [33, 42].

### HHT derived from *Cephalotaxus*

HHT is a new anti-leukemia drug that is recommended for clinical application and was first recommended in China [43]. Chinese people have used *Cephalotaxus* to treat tumors for a long time. HHT is derived from *Cephalotaxus*, and it is a natural plant alkaloid with anti-tumor activity [44]. HHT inhibits protein synthesis in eukaryotic cells. In addition to its anti-tumor activity, HHT induces leukemia differentiation and maturation and promotes leukemia cell apoptosis. After intravenous injection, the highest concentration of HHT was found in the bone marrow, followed by the kidneys, liver, spleen, heart, and gastrointestinal tract, while the lowest concentration was found in muscle and brain tissue. After 2 h of intravenous administration, the drug concentration in all tissues decreased rapidly, while the concentration in the bone marrow decreased more slowly [45].

The clinical application of HHT has proven efficacious. In the early years, the use of the HA regimen (HHT combined with cytosine arabinoside, Ara-C) for the treatment of AML achieved significant efficacy, and HHT has demonstrated no cross-resistance with Daunorubicin (DNR), Ara-C, and mercaptopurine [46, 47]. HHT is recommended in the consolidation phase in the current APL diagnosis and treatment guidelines in China [3, 20]. A clinical study showed no significant difference in the treatment efficacy of HHT and DNR in patients with APL, but HHT-treated patients had a better quality of life [48]. Another retrospective study showed that induction therapy with ATRA-HHT for APL resulted in a CR rate of up to 100%, with no deaths during induction therapy, and 9-year DFS and overall survival rates of 79.0% and 83.0%, respectively. Moreover, this regimen was well tolerated and hepatotoxicity could be recovered therapeutically. No serious cardiac or renal toxicity was observed, and no secondary tumors occurred during the follow-up period. Therefore, this regimen may be a highly effective therapeutic option for patients with newly diagnosed APL [49]. The reasons for choosing HHT go beyond its efficacy. A clinical trial of HHT in combination with ATRA and ATO for the treatment of economically disadvantaged patients with initial APL demonstrated lower healthcare costs in the HHT group due to fewer blood transfusions and a lower incidence of infections compared with the commonly used Idarubicin (IDA). HHT also has fewer cardiotoxic side effects in pediatric patients, elderly patients, and patients with a history of cardiac disease [50]. HHT in combination with other medications for the treatment of APL is effective and safe, and it is therefore one of the choices in the clinic.

## Natural medicines with therapeutic potential for APL

Although ATRA and ATO are well-established treatment options for APL, the occurrence of various adverse effects still seriously affects the quality of life of patients. ATRA induces terminal differentiation of leukemia cells and significantly improves the prognosis of patients with APL. However, the continued use of drugs, such as ATRA, usually causes significant treatment-related toxicity, resulting in ATRA resistance, retinoic acid syndrome (RAS), hypercalcemia, and decreased plasma drug concentrations [51]. Natural drugs are usually characterized by low toxicity and multi-pathway action, and they are more biocompatible than chemical drugs. Therefore, natural products can be used as a potential complementary resource to chemotherapeutic drugs.

### Small-molecule compounds with therapeutic potential for APL

#### Honokiol

**Fig. 3:**
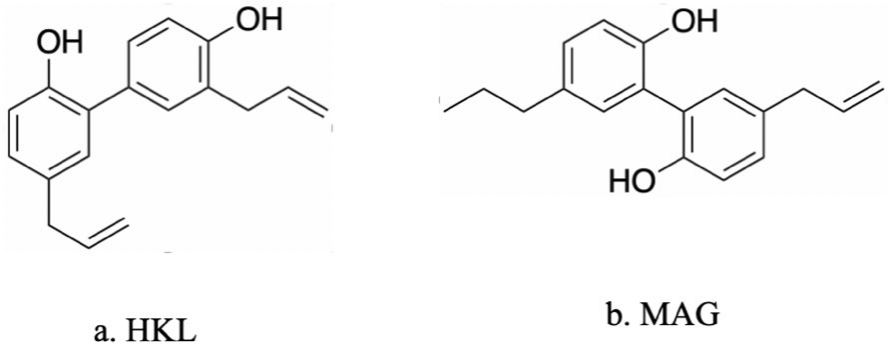
Structure of HKL and its analogue MAG

*Magnolia officinalis* and *Magnolia obovata* bark extracts have been utilized as traditional medicines in China and Japan for centuries. These extracts are commonly employed in traditional medicine to address various health conditions, offering sedative, antioxidant, anti-inflammatory, antibiotic, and spasmodic effects. Additionally, they exhibit significant anticancer potential while maintaining low toxicity towards healthy cells. Honokiol (HKL) and magnolol (MAG), as shown in Fig. 3, are natural lignans with multiple effects that can be extracted from *Magnolia grandiflora*. Their safety and efficacy have gained widespread recognition [52, 53].

Although both HKL and MAG are active ingredients extracted from *Magnoliaceae*, NB4 cells are more sensitive to HKL than MAG, which significantly reduces the activity of NB4 cells. Interestingly, the pathway of HKL-induced NB4 cell death does not involve apoptotic features, such as caspase activation and nucleus fragmentation, and its apoptosis-inducing process is accompanied by increased reactive oxygen species (ROS), mitochondrial damage, and expansion of the endoplasmic reticulum by triggering the accumulation of misfolded and unfolded proteins. This induces extensive cytoplasmic vacuolization and NB4 cell apoptosis. As cancer cells can evade apoptotic cell death through a variety of adaptive mechanisms, HKL, which induces cancer cell death in a non-apoptotic manner, could be an important drug for the treatment of APL [54].

In addition to inducing apoptosis, HKL is effective when used in combination with other therapies. In the treatment of myeloid leukemia, the combination of HKL with low-concentration chemotherapeutic agents has significant synergistic cytotoxic effects, which can effectively reverse drug resistance and reduce drug toxicity [55]. Moreover, HKL has a significant synergistic effect with cytarabine for the treatment of AML, where it inhibits cell proliferation and induces apoptosis [56]. However, the effect of this regimen on APL has not been confirmed. In the treatment of APL, HKL counteracts the toxic effects of ATO on the cardiac mitochondria and exerts cardioprotective effects against ischemia/reperfusion chemistry-induced cardiotoxicity. This result was confirmed in a mouse model, in which mice pretreated with HKL demonstrated significant amelioration of ATO-induced myocardial apoptosis, cardiac dysfunction, and cardiac remodeling [57]. Therefore, the combination of ATO and HKL for the treatment of APL may achieve good safety.

#### Sesquiterpene lactones

**Fig. 4:**
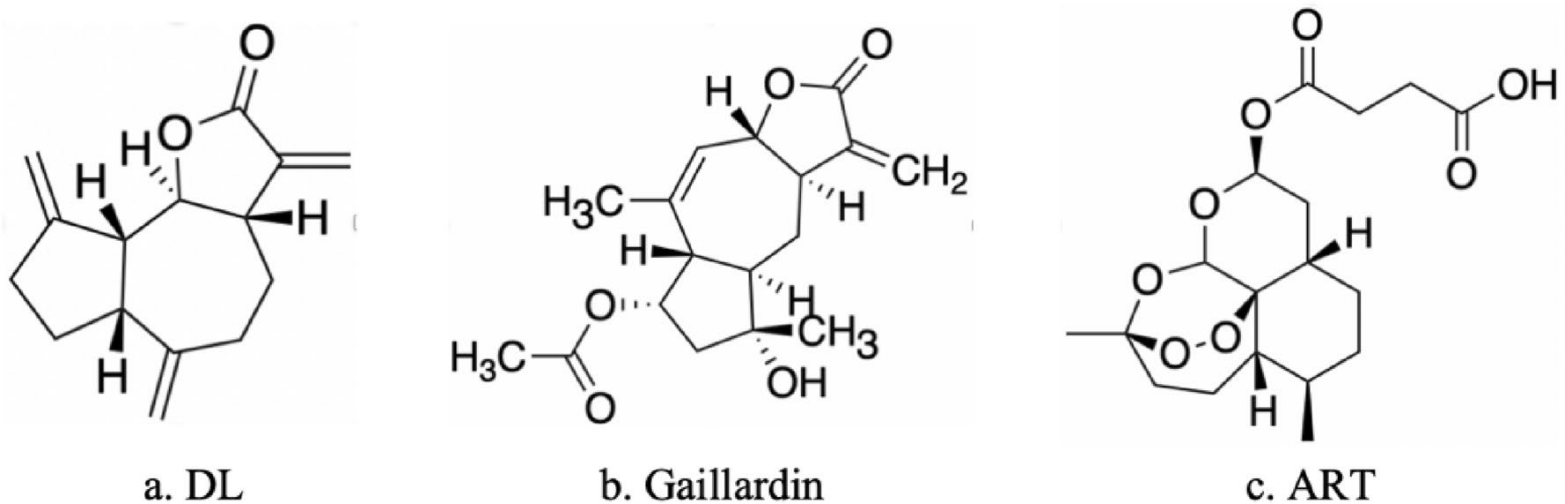
Chemical structures of the sesquiterpene lactone compounds gaillardin and artesunate (ART), which consist of three isoprenoid units and usually have multiple biological properties

Sesquiterpene lactones are a class of secondary metabolites. They are a large group of naturally occurring compounds with a wide range of notable biological properties, such as anti-inflammatory, anti-bacterial, and anti-tumor properties. Sesquiterpene lactones are mostly derived from the *Compositae* family, and families such as *Cactaceae*, *Solanaceae*, and *Euphorbiaceae* may also contain sesquiterpene lactones. Many of the active constituents of traditional medicinal plants used for various ailments, such as infections, inflammation, and headaches, contain sesquiterpene lactones [58]. Their main anti-tumor mechanisms include oxidative stress, iron death, induction of apoptosis, and cellular autophagy. *Aucklandia lappa* Decne. from the *Asteraceae* family, which has been used in the combinatorial treatment of leukemia in Xiangsha Six Gentlemen Decoction, contains the sesquiterpene lactone compound dehydrocostus lactone (DL). DL can enhance TNF-α-induced apoptosis and has anti-leukemia activity in vitro [59]*.* The natural products of sesquiterpene lactones not only inhibit drug-resistant tumor cells, but also present sensitizing and potentiating effects when used in combination with other drugs. However, current research on drug-resistant cells and drug combinations is still limited [58]. Sesquiterpene lactones, such as gaillardin and artesunate, also show therapeutic potential for APL. Chemical structures of the sesquiterpene lactone compounds DL, gaillardin, and artesunate are shown in Fig. 4.

#### Gaillardin

*Inula* sesquiterpene lactones are a kind of sesquiterpene lactones extracted from *Inula* species. They have many pharmacological activities such as anti-inflammation, anti-asthma, anti-tumor, neuroprotection, and anti-allergy. The *Inula* genus has long been used in folk medicine to treat various ailments including kidney stones, urethral infections, jaundice, bronchitis, respiratory diseases, and cancer. It is widely utilized as a traditional medicine across Asia, the Middle East, Europe, and North America. In recent years, numerous studies have increasingly demonstrated the significance of these drugs as potential candidates for treating various types of cancers due to their strong anti-tumor activity [60–62]. Gaillardin, a sesquiterpene lactone isolated from the chloroform extract of *Inula oculus-christi* L., is toxic to a variety of cancer cells. It has been demonstrated that gaillardin induces cytotoxicity through the G0/G1 phase blockade and then apoptosis in a dose-dependent manner and that it has no significant cytotoxic effect on healthy cells, making it a promising anti-hematological malignancy medicine that could open new avenues for the treatment of APL [63].

In vitro experiments further support this idea. In a previous study, gaillardin dose-dependently induced apoptosis in APL cells. Gaillardin extracts at concentrations of 1, 4, and 5 μM induced early apoptosis in 10.5%, 19%, and 32% of NB4 cells, respectively, with an IC_50_ of approximately 7 μM after 48 h. Treatment of NB4 cells with gaillardin resulted in the upregulation of Bax transcripts and a decrease in Bcl-2 mRNA, in turn increasing the Bax/Bcl-2 transcription ratio. At the tissue level, Bcl-2 and Bax dimers formed, which initiated the release of cytochrome C from the mitochondria and activated caspase-3, ultimately leading to cell death [64]. In vivo experiments evaluating the treatment of APL with gaillardin are expected.

#### Artesunate

Artemisinin analogs, with their unique peroxy-bridge structure, have been shown to have significant therapeutic effects against *Plasmodium falciparum*, which causes malaria, and are not susceptible to drug resistance [65]. One of the herbs in the Chinese prescription Qinggu Powder is *Artemisia annua* L. In leukemia, artemisinin has been shown to induce cell cycle arrest [66]. Artesunate (ART) is a semi-synthetic derivative of artemisinin, which has the advantages of oral administration and good water solubility. It is widely used clinically for the treatment of malaria and has shown anti-tumor effects against a variety of hematologic tumors. Studies have shown that, after the treatment of NB4, HL-60, and NB4-R1 (retinoic acid-resistant strains) with 2, 10, or 20 μg/mL ART for 12, 24, or 48 h, cell proliferation was significantly inhibited in an obvious time- and concentration-dependent manner and the cells showed typical apoptotic morphology changes after 24 h. The mechanism of action of ART may be phosphorylation of the JNK pathway in the form of p-JNK, p-MKK4, and p-ATE-2, as well as inhibition of PI3K/AKT/mTOR pathway phosphorylation [67]. ART shows potential for the treatment of APL and retinoic acid-resistant APL, but its therapeutic efficacy needs to be further demonstrated.

#### Celastrol

**Fig. 5:**
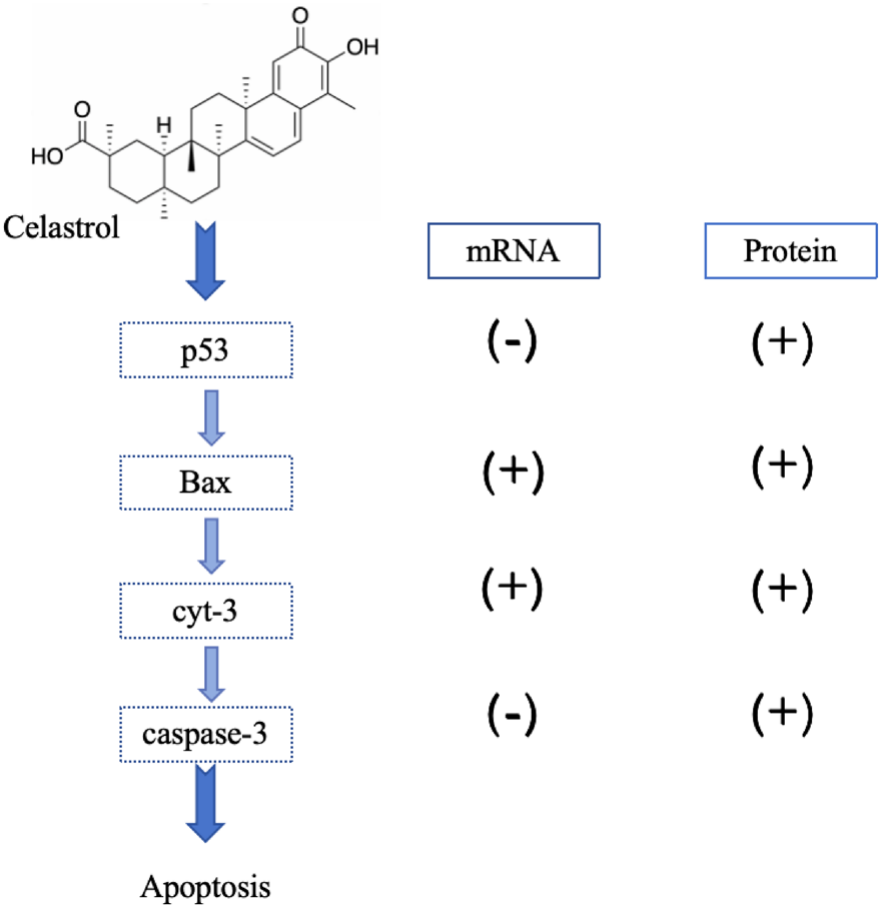
Celastrol induces apoptosis in APL cells through the mitochondrial pathway, causing changes in cytokines

Celastrol is a natural pentacyclic triterpenoid purified from the Celastraceae family. Celastrol possesses a variety of properties as a TCM, including anti-inflammatory and broad-spectrum anti-cancer properties [68]. Celastrol achieves its anti-malignant properties against hematological neoplasm through several pathways. First, celastrol is a potent low-molecular-weight inhibitor that induces myeloid differentiation and cancer cell apoptosis by inhibiting Myb activity. In combination with other compounds, the inhibitory effect of celastrol on cell proliferation can be enhanced [69]. Second, several studies have proven that celastrol can induce apoptosis in APL cells through the p53-activated mitochondrial pathway [70]. As shown in Fig. 5, the mRNA expression of caspase-9, caspase-3, and Bax was elevated after celastrol treatment, while the mRNA expression of p53 was not. The protein expression of cleaved caspase-9, cleaved caspase-3, Bax, and p53 was significantly elevated. After 24 and 48 h, inhibition of HL-60 cell proliferation occurred in a dose-dependent manner, with IC_50_ values of 0.48 and 0.55 μM at 24 and 48 h.

Celastrol has a satisfactory safety profile in the treatment of APL. In the nude mouse model of APL with tumor xenografts, there was no significant difference in the coefficients of the heart, liver, spleen, lungs, kidneys, brain, testes, and epididymides between the control and celastrol-treated groups of mice. There were no statistically significant differences between the alanine transaminase (ALT), aspartate transaminase (AST), blood urea nitrogen (BUN), and creatinine concentrations (CREA) of control mice and celastrol-treated mice, which were all within normal ranges, and no obvious histopathology was seen in the testes of mice, suggesting that celastrol has no toxic effects on the liver, kidneys, or reproductive system at a dose of 2 mg/kg [70]. Additional in vitro results showed that the ability of leukemia cells from two different AML mouse models to form colonies in the semisolid medium was inhibited by sub-micromolar concentrations of celastrol, but the proliferative capacity of normal hematopoietic progenitor cells from healthy mice was not inhibited under the same conditions. Similarly, colony formation assays performed on leukemic cells from patients with AML and cells from healthy donors confirmed that leukemic cell proliferation was significantly inhibited by celastrol, whereas healthy progenitor cells were unaffected [69]. These results suggest that celastrol has a good safety profile in the treatment of leukemia and is a useful alternative combination therapy.

#### Tanshinone IIA

*Salvia miltiorrhiza* Bunge is an important component of oral arsenic agent RIF. Tanshinone IIA (Tan IIA) is a diterpene quinone isolated from *Salvia miltiorrhiza* Bunge, which is the most abundant and structurally representative fat-soluble constituent of *Salvia miltiorrhiza*, and has been found to possess anti-tumor properties, such as inducing cell autophagy and apoptosis, and inhibiting tumor invasion and metastasis [71]. Many studies have proven that Tan IIA has the biological activity to treat APL by inducing apoptosis in the APL cell line NB4 [72]. It has also shown excellent therapeutic effects when combined with ATRA and ATO.

**Fig. 6:**
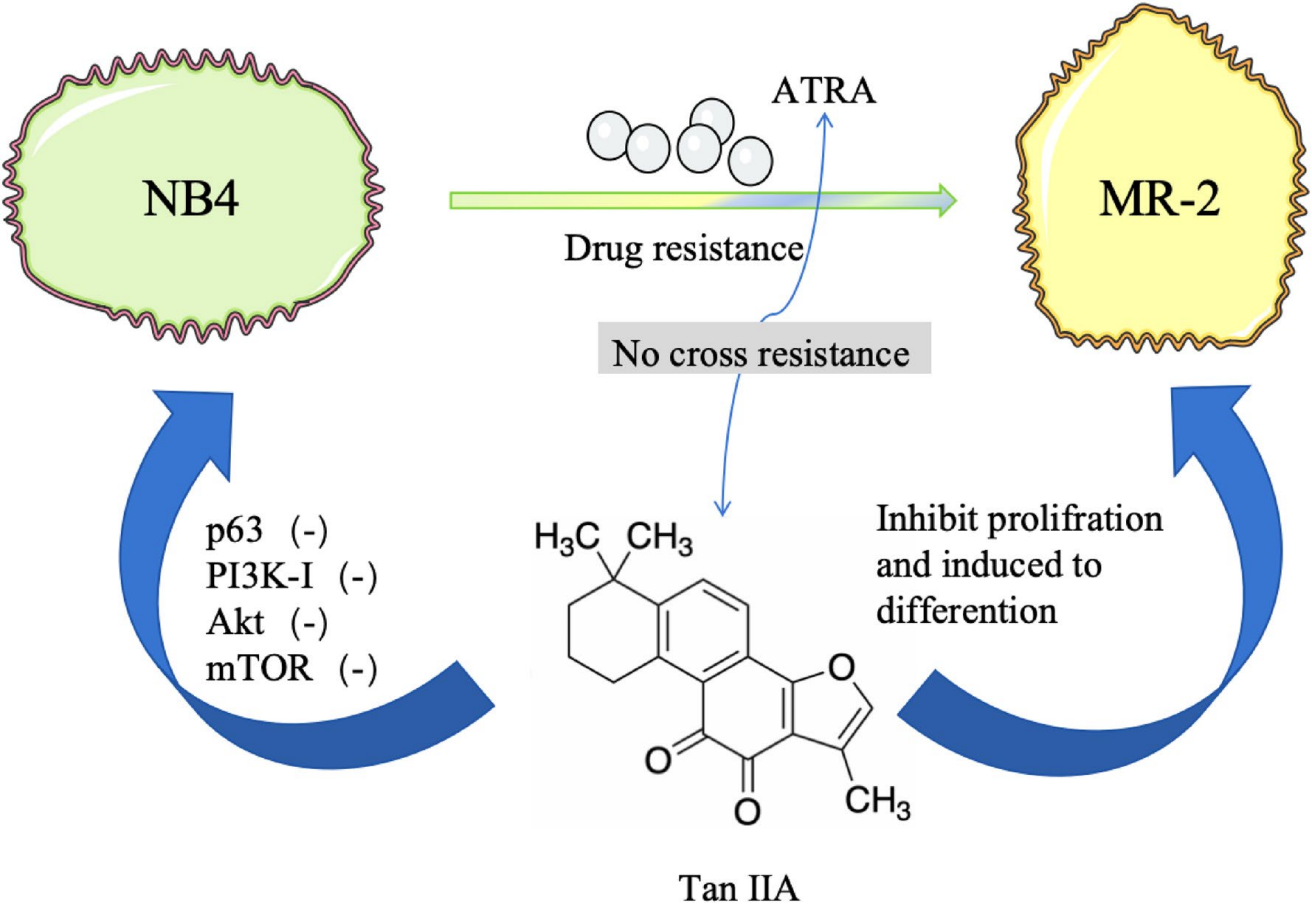
Tan IIA is therapeutically effective against both NB4 and MR-2, without cross-resistance

In a previous study, Tan IIA inhibited the growth of NB4 cells and induced the differentiation of NB4 cells, and these effects were gradually enhanced with an increase in drug concentration and a prolonged duration of action. Similar to As_2_O_3_ and ATRA, Tan IIA did not change the expression of *PML-RARα* mRNA, but it degraded the *PML-RARα* fusion protein and restored the expression of the PML protein. The optimal concentration to achieve this effect was 2.55 μmol/L [73]. In addition, Tan IIA induced NB4 cell autophagy to form the autophagic stream in a concentration- and time-dependent manner. The effects of Tan IIA on NB4 cells, evaluated after 48 h., included significantly reduced expression of the autophagy-related protein p62, and increased autophagy rate of NB4 cells. PI3K-Akt, and mTOR protein bands in NB4 cells were less pronounced than those in the control group after treatment with Tan IIA, which indicated that Tan IIA reduced the expression of the PI3K-I, Akt, and mTOR proteins in NB4 cells. In short, Tan IIA reduced the Akt and mTOR phosphorylation levels, and inhibited PI3K/Akt/mTOR signaling [74]. Thirdly, when Tan IIA was combined with ATO, the apoptosis and autophagy rates of NB4 cells were higher than those of the single-drug group. This may be due to the fact that Tan IIA-ATO can upregulate the expression of the apoptosis-specific protein caspase-3 and the autophagy-specific protein LC3-II in transplanted tumor tissues, as well as enhancing tumor cell apoptosis and autophagy. Therefore, the Tan IIA-ATO combination has a chemo-sensitizing effect on NB4 transplanted tumors. In addition, experiments have shown that the Tan IIA-ATO regimen causes no obvious pathological damage to the bone marrow, heart, liver, lungs, kidneys, lymph nodes, and other important tissues and organs in nude mice, demonstrating its safety [75]. Fourth, as shown in Fig. 6, Tan IIA still has a therapeutic effect in ATRA-resistant strains. MR-2 is an ATRA-resistant APL cell type. When Tan IIA was co-cultured with NB4 and MR-2 cells, Tan IIA caused differentiation and apoptosis of both cell types, which indicated that there was no cross-resistance between ATRA and Tan IIA. Tan IIA at 1.0 mg/L inhibited the proliferation of MR-2 cells and induced their transformation into granulocytes, which is the most effective way to prevent the proliferation of ATRA [76]. Tan IIA at 1.0 mg/L inhibited the proliferation of MR-2 cells and induced their differentiation to the mature stage of the granulosa lineage, and the effect was comparable to that of 0.5 mg/L Tan IIA, which induced the differentiation of NB4 cells [77]. From the above studies, it is clear that Tan IIA may be a promising clinical treatment for APL, especially for recurrent and drug-resistant patients.

#### Oleanolic acid and its derivatives

Oleanolic acid (OA), a pentacyclic triterpenoid that is ubiquitous in the plant kingdom, is the main active ingredient of *Akebia quinate* (Thunb.) Decne. in Longdan Xiegan Decoction, *Forsythia suspensa* (Thunb.) Vahl in Lianhua Decoctio and *Hedyotis diffusa.* OA has been endowed with an extensive variety of biological properties and therapeutic potential through its complex and multi-factorial mechanisms, and it has received much attention from the scientific community because of its biological activity in a wide range of diseases. OA and related triterpenes have a wide range of pharmacological properties, but their therapeutic potential has only been partially exploited to date [78]. The anti-cancer potential of bioactive triterpenes in vitro and in vivo models, including in the treatment of APL, has been widely discussed [79].

OA enhances the differentiation of APL cells and prevents the development of leukemia in mice [80]. Firstly, OA and its analog ursolic acid (UA) significantly inhibited the proliferation of HL-60 cells in a concentration- and time-dependent manner from 0 to 72 h after treatment. The number of non-living cells was higher for cells cultured at high OA and UA concentrations of 80–100 μM. At non-cytotoxic concentrations, OA had a more significant differentiation-inducing effect on HL-60 cells, and when combined with low-dose ATRA, OA increased the differentiation rate of HL-60 cells, whereas UA had no significant effect on the differentiation of ATRA. In a mouse model of leukemia, OA increased the survival time and decreased the infiltration of leukemia cells into the liver and kidneys.

**Fig. 7:**
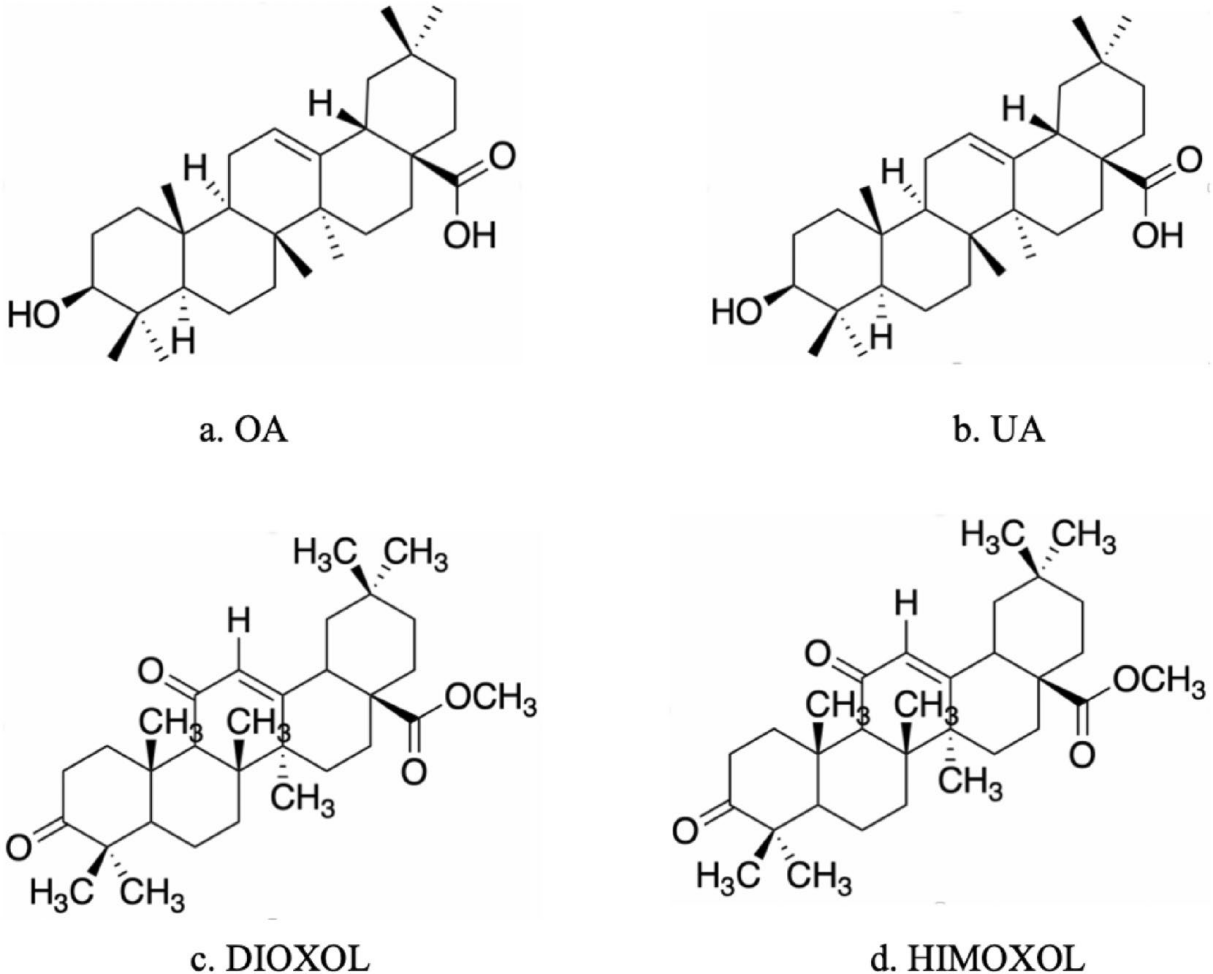
Structure of OA and its derivatives, which all have therapeutic potential for APL

The structures of OA and its derivatives are shown in Fig. 7. The OA derivatives DIOXOL and HIMOXOL may also be therapeutically effective against HL-60 cells, its overexpressing subline HL-60/AR, and its multidrug-resistant subline ABCC1. DIOXOL and HIMOXOL are the most potent semi-synthetic OA derivatives against human APL cells [81]. Cell cycle analyses of 5–20 μM DIOXOL and HIMOXOL treatment for 24 h showed the presence of sub-G1 cell populations, indicating DNA fragmentation of dead cells. Among them, DIOXOL was the most effective at inducing apoptosis in HL-60 cells, and higher concentrations of DIOXOL (10 μM and 20 μM) activated apoptosis to a greater degree than HIMOXOL. DIOXOL significantly reduced p65 nuclear factor kappa-B (NF-κB) and inhibited its translocation to the nucleus to activate the apoptotic program. A 70% reduction in intracellular NF-κB subunit content was observed in samples treated with 20 μM DIOXOL. HIMOXOL is the most effective compound against drug-resistant HL-60/AR cells. It can inhibit ABCC1 transporter function in a short period and reduce ABCC1 protein expression over a longer period. HIMOXOL at concentrations of 5 and 10 μM were able to act at the transcriptional level, leading to significant reductions in ABCC1 transcripts of approximately 30% and 50%. HIMOXOL was also more effective at reducing the amount of Bcl-2. Bcl-2 was reduced by 15% when HIMOXOL was used at a concentration of 10 μM, which was increased to 70% when HIMOXOL was used at a concentration of 20 μM. OA and its derivatives could be used as part of an initial screen of potential synergistic anti-leukemic agents for ATRA, providing a direction for new APL drug development.

### Active extracts from plants

#### Natural seaweed extracts—fucoidan

Fucoidan is a high molecular-weight, fucose-based, sulfated polysaccharide extracted from the brown macroalgae. It is a natural component of seaweed and is found in the cell walls of a range of brown seaweeds. Fucoidan is a heterogeneous sulfated polysaccharide containing sulfated L-fucose with 34–44% fucose content, which has immunomodulatory and anti-tumor effects [82, 83].

In a previous study, fucoidan inhibited the proliferation and induced the apoptosis of the APL cell lines NB4 and HL-60 via both endogenous and exogenous pathways. The proliferation of NB4 and HL-60 cells was inhibited in a dose-dependent manner, and the cell proliferation of HL-60 and NB4 cells decreased to less than 10% at fucoidan concentrations of 50 and 25 μg/mL, respectively. After treatment of NB4 and HL-60 cells with 100 μg/mL fucoidan for 48 h, the percentage of sub-G0/G1 cells in the dead cell population increased significantly in a time-dependent manner, and fucoidan significantly increased apoptosis in both cell lines. After 10 days of inoculation of NB4 cells into seven nude mice in each of the two groups, five in the control group developed subcutaneous tumors, whereas only two in the fucoidan group developed subcutaneous tumor masses, and no other toxicity was observed in either group [84]. These findings collectively suggest that fucoidan significantly delays tumor growth.

The conventional therapy for APL is ATRA-ATO, but drug resistance or RAS may occur with long-term use of this regimen. Some findings suggest that, by adding fucoidan to the standard APL regimen, the number of resistant cells in patients who respond to ATRA can be limited [85]. Fucoidan combined with the ATRA-ATO regimen synergistically induced NB4 cell differentiation, as evidenced by increased CD11b expression and G0/G1 blockade. In vitro findings showed that a portion of cells remained undifferentiated when cells were treated with ATRA alone or ATRA-ATO, whereas almost all cells underwent differentiation when fucoidan was combined with ATRA-ATO. CD44 expression in APL cells was reduced when mouse tumor cuts were treated with fucoidan combined with ATRA, implying that the use of this regimen may decelerate the spread of cancer cells in patients with APL. The use of fucoidan as a supplement to standard APL therapy may represent a promising new strategy for APL management.

#### Crocin and crocetin from saffron

**Fig. 8:**
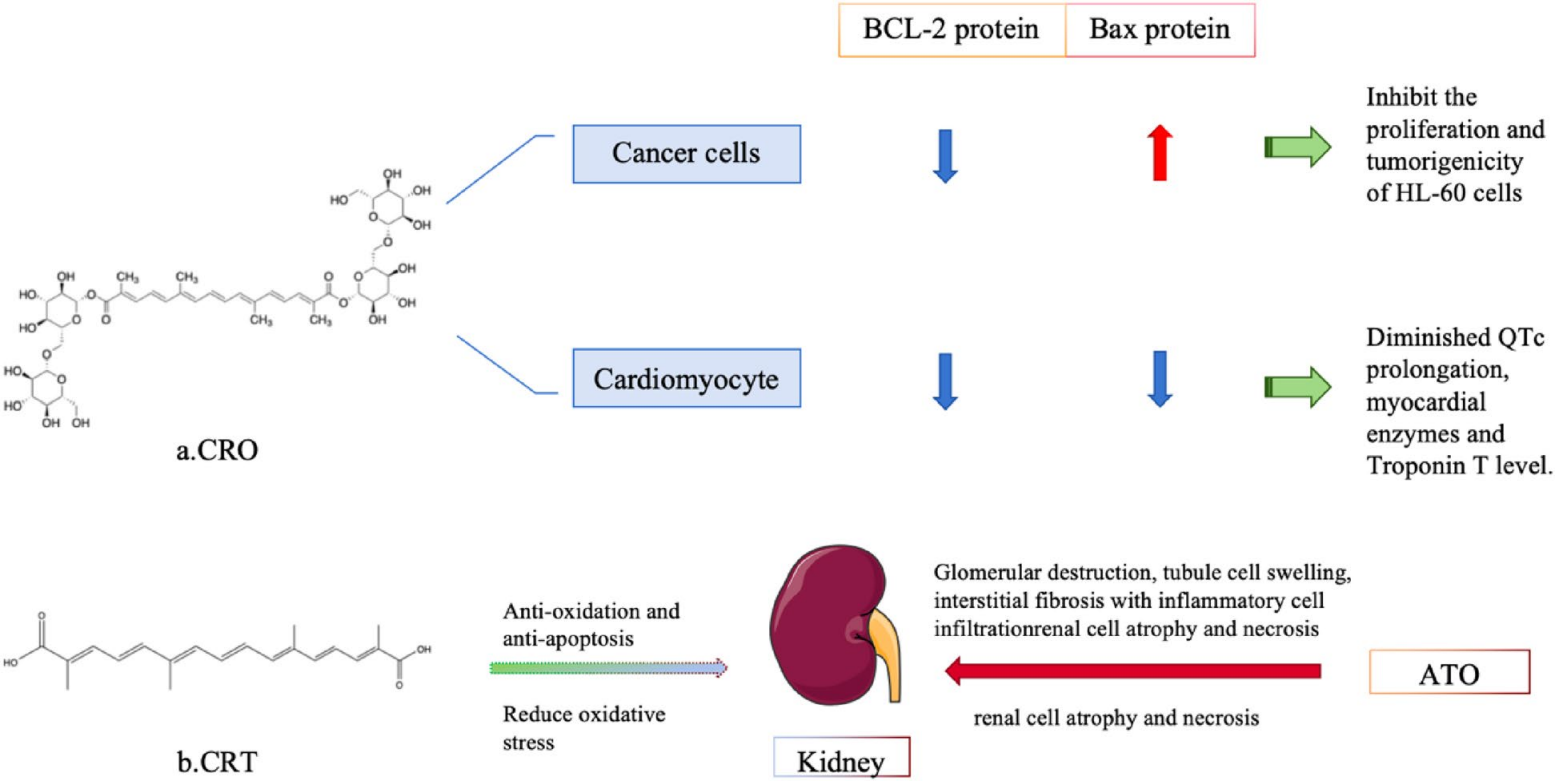
The bioactive substances CRO and CRT in saffron. CRO inhibits the proliferation and tumorigenicity of HL-60 cells. CRT has anti-oxidant and anti-apoptotic properties and can significantly reduce oxidative stress in ATO-induced nephrotoxicity

Saffron is derived from *Crocus sativus* L., which is a valuable medicinal plant in many traditional medicinal cultures. There are around 75 species of crocus in the world, and saffron is the only species available for medicinal use. It is mainly distributed in Southern Europe and Iran, and also planted in China [86]. Saffron has more than 200 active ingredients, including pigments, flavonoids, phenolic acids, and fatty acids, amongst others [87]. *Gardenia jasminoides* Ellis, commonly used in TCM, is one of the components of Longdan Xiegan Decoction, and also contains similar components. *The Compendium of Materia Medica* describes saffron as “The smell is sweet, flat, and non-toxic. Indications: Heart worries and stagnation, persistent *Qi* stagnation, and promoting blood circulation. Long-serving brings joy and treats palpitations.” Among the active ingredients, crocin (CRO) and crocetin (CRT) have great potential for the treatment of APL. The activity mechanism of CRO and CRT is shown in Fig. 8.

CRO is the main water-soluble carotenoid in saffron extract. It has anti-tumor activity against many human tumors [88]. Studies have shown [89] that CRO at a certain concentration range (0.625–10 mg/mL) significantly inhibits the proliferation of HL-60 cells, and with an increase in the CRO concentration from 0.625 to 5 mg/mL, the percentage of apoptotic cells increases significantly, and this effect is time-dependent. In the nude mouse HL-60 cell model, the tumor formation time in the experimental group (6.25 mg/kg CRO) was significantly longer than in the other groups, and the tumor formation time in the experimental group (25 mg/kg CRO) was longer than in the control group and the experimental group (100 mg/kg CRO). Compared with the control group, the rate of change in tumor weight and tumor size was significantly suppressed in mice treated with 6.25 and 25 mg/kg CRO. Moreover, Bcl-2 protein expression was reduced and Bax protein expression was elevated in the tumor. The above findings prove that CRO inhibits the proliferation and tumorigenicity of HL-60 cells.

In addition to its therapeutic potential, CRO in combination with ATO reduces ATO-induced cardiotoxicity [90]. CRO administration not only reduces QTc interval prolongation, cardiac enzymes, and troponin T, but it also improves histopathological results. The expression of Bax and caspase-3 in the myocardium of rats treated with CRO was significantly decreased compared to when rats without CRO. CRO appears to reduce ATO-induced myocardial pathological changes, and the therapeutic effect of CRO appears to be dose-dependent. Similarly, CRT may be protective against ATO-induced renal injury [91]. CRT has anti-oxidant and anti-apoptotic properties, and therefore it can significantly reduce oxidative stress in ATO-induced nephrotoxicity. In one study, ATO-induced histopathological changes in the kidneys of rats showed glomerular destruction, tubular cell swelling, interstitial fibrosis with inflammatory cell infiltration, and nephrocyte atrophy and necrosis. Treatment with 25 or 50 mg/kg CRT significantly reduced the morphological changes in the kidney induced by ATO. From the above, it is reasonable to propose that CRO and CRT are ideal choices as combined treatments with ATO, and the usefulness of these combinations should be further investigated for clinical application.

#### Green tea extract

**Fig. 9:**
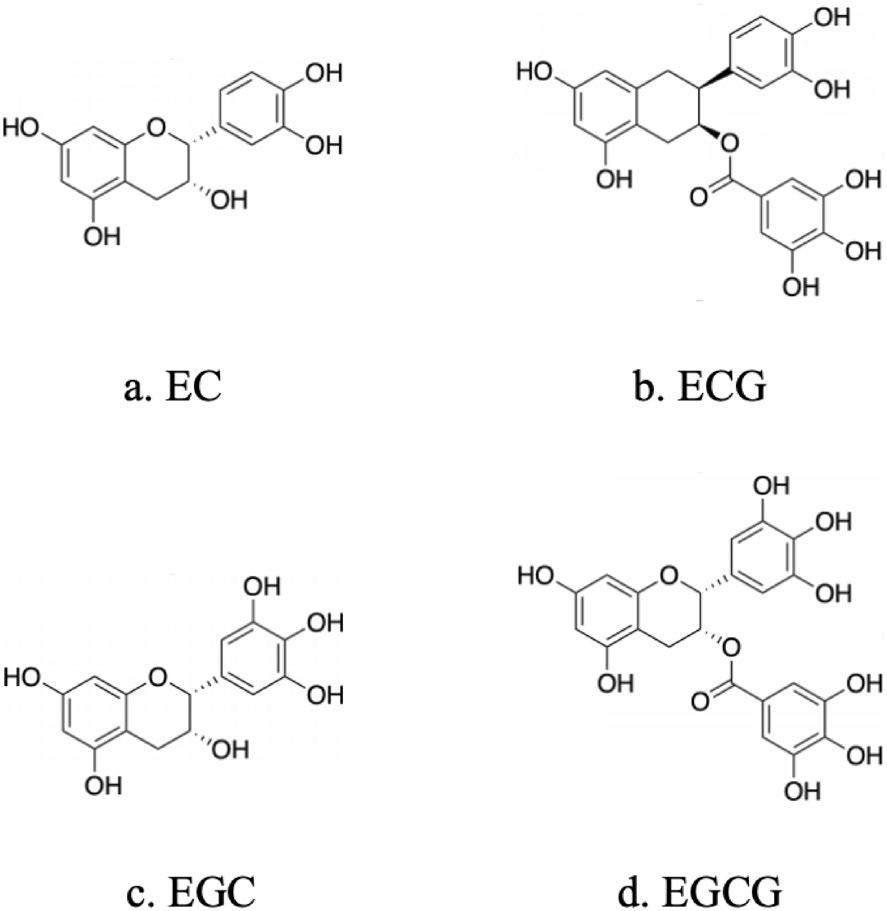
The four main active substances of catechins: EC, ECG, EGC, and EGCG

Tea is one of the most popular drinks in the world. Originating from China, tea was introduced to the world thousands of years ago via the Silk Road. The production of green tea involves decoction or steaming of freshly harvested leaves to inactivate polyphenol oxidase and other enzymes that prevent fermentation/oxidation, preserving the active chemical properties [92]. As one of the most consumed beverages worldwide, green tea has been the focus of much research, and its polyphenolic compounds have been shown to have many benefits for human health. Catechins are the main components extracted from green tea leaves and are present in about 30% of dried green tea, which includes epigallocatechin gallate (EGCG), epicatechin gallate (ECG), epigallocatechin (EGC), and epicatechin (EC), as shown in Fig. 9. Catechins are inexpensive, safe, and can be administered orally. Catechins, especially EGCG, have multifaceted effects that make them attractive candidates for the prevention and treatment of leukemia and myelodysplastic syndrome [92, 93].

A previous study showed that green tea extract reduced leukocytes and immature cells (progenitor cells) in the peripheral blood, bone marrow, and spleen of leukemic mice while increasing mature cells in the bone marrow. An important observation in leukemic mice is an increase in the number of leukocytes, and treatment with 250 mg/kg green tea extract for 4 days decreased the percentage of leukocytes while decreasing the percentage of immature cells and increasing the percentage of mature cells. These results suggest that green tea extract has anti-leukemic proliferative effects in vivo by inhibiting malignant clonal expansion [94]. In addition, catechins can have anti-leukemic activity by inducing apoptosis. In another study, NB4 cells were inoculated subcutaneously in nude mice, and 10 mM catechin was used as the only drinking water of the mice for 10 days. Tumor size was significantly reduced in the treated group, and no tumor infiltration was detected in any organ at necropsy. The *PML-RARα* fusion protein was degraded after treatment of primary leukemia cells with 100 and 150 μM catechin for 24 h [93]. This is a strong rationale supporting the therapeutic potential of catechins for APL.

Of the four major catechins, EGCG is the most abundant and potent polyphenolic compound in green tea extract, accounting for 50–75% of total catechins [92]. Due to its long half-life, the compound is rapidly absorbed and distributed in all tissues [93]. EGCG has emerged as an effective inducer of apoptosis through mechanisms involved in caspase activation, regulation of the Bcl-2 family of proteins, disruption of survival signaling pathways, and modulation of redox balance and induction of oxidative stress [92]. It has been shown that EGCG can specifically cause tumor cell death, but it is not toxic to healthy cells. Exposure of HL-60 cells to EGCG reduced cell proliferation and induced apoptosis. When HL-60 cells were treated with EGCG, there was a time-dependent decrease in cell proliferation, and significant induction of apoptosis was seen in EGCG-treated HL-60 cells at day 9 [95]. With the combination of ATRA and EGCG for the treatment of myeloid leukemia, neutrophil differentiation was enhanced. In combination with ATRA, the ATRA-induced effect of DAPK2 activation was enhanced, and granulocyte maturation was enhanced [96]. In addition, as a cell proliferation inhibitor and epigenetic modifier, EGCG may be useful for the treatment of APL [97].

#### Crude methanolic extract of *Mucuna macrocarpa*(CMEMM)

*Mucuna macrocarpa* Wall. (*Leguminosae*) is a large woody climber that is distributed in China, mainly in Yunnan, Guizhou, Guangdong, Hainan, Guangxi, Taiwan, and Southeast Asia. In folk medicine, the dried stems of this species have been used to enhance blood circulation in a variety of hematological and circulatory disorders [98].

CMEMM in combination with ATO increases the efficacy of ATO [98]. Specifically, HL-60, Jurkat, and Molt-3 cells were treated with 2.5 or 5 μM ATO alone or in combination with increasing doses of CMEMM (25–75 μg/mL). It was found that CMEMM enhanced the growth inhibition of HL-60, Jurkat, and Molt-3 cells after ATO treatment for 24 or 48 h, and combined treatment with ATO and CMEMM synergistically inhibited the growth of HL-60 cells. Additionally, apoptotic morphology and flow cytometry data indicated higher apoptosis induction with combined treatment than with ATO or CMEMM alone. Further studies showed that leukemia cell apoptosis induced by ATO-CMEMM was mediated by oxidative stress [99]. Compared with ATO alone, higher levels of cleaved caspase-3, caspase-9, and poly (ADP-ribose) polymerase were present in HL-60 and Jurkat cells exposed to the ATO*-*CMEMM combination. In addition, the in vitro antiproliferative effect of 25–75 μg/mL *Mucuna macrocarpa* on HL-60 cells was dose-dependently and time-dependently enhanced at 72 h. The IC_50_ of CMEMM was 36.4 μg/mL after 7 h of exposure, and the cells showed apoptotic characteristics. CMEMM (500 mg/kg/day by intraperitoneal injection) inhibited tumor growth in mouse xenografts in vivo. CMEMM exerts its anti-leukemic effect in HL-60 cells through the apoptotic pathway, and it may be considered an anti-leukemic drug candidate in the future.

## Discussion

### Natural products have good prospects for the treatment of malignant tumors

Nature has always been the natural pharmacy for mankind. Before the advent of modern science and technology, human beings obtained medicines from nature to treat their diseases. The development of modern science and technology is also accompanied by a growing use of traditional medicines worldwide. Natural medicines usually have multiple targets and pathways of action; therefore, they have a variety of biological properties for the treatment of a wide range of diseases, which means that they are often preferred over single-target drugs. In the first two decades of the twentieth century, coronaviruses have ravaged the world several times. Multi-target natural medicines have played an important role in human resistance to coronavirus [100]. Nowadays, although various therapies are emerging for the treatment of malignant tumors, natural medicines are not to be ignored because of mild, safe, and inexpensive.

Natural medicines have good clinical evidence in the treatment of malignant tumors. Paclitaxel, an active ingredient derived from *Taxus cuspidata* Sieb. Et Zucc., has been widely used in the clinic as a broad-spectrum drug to treat diseases such as breast cancer [101]. In the treatment of hematological malignancies, vincristine, a dimeric indole alkaloid from the leaves of *Catharanthus roseus* (L.) G. Don, is used in the treatment of acute lymphoblastic leukemia [102], Hodgkin’s lymphoma [103], and other hematological malignancies. Podophyllin is a natural active ingredient derived from the *Dysosma* species, and its derivative etoposide is a highly active anti-tumor drug, which is used in the treatment of hemophagocytic syndrome [104, 105]. ATO, which is derived from natural products, has become the first-line drug for the treatment of APL worldwide, and the combination of ATO and ATRA can achieve an APL remission rate of 90%. However, this regimen is prone to toxicity and side effects, such as drug resistance, RAS and cardiotoxicity with prolonged use, so it is essential to adopt a combination of drugs for treatment. In addition to oral RIF, which can reduce the cardiotoxicity of arsenicals, natural drugs, such as HKL, CRO, and HHT, can protect the heart when used in combination with ATO. Natural drugs, such as ART, Tan IIA, OA and its derivatives, and fucoidan, amongst others, affect ATRA-resistant cells; therefore, they may be useful therapeutic options in the clinic. In addition to the natural drugs mentioned in this paper, other natural products, such as *Acanthopanax senticosus* Harms leaf extract [106], *Patrinia heterophylla* Bunge [107], Korean red ginseng extract [108], and quercetin [109], amongst others, also have therapeutic potential for treating APL, which should be further explored.

Cancer cells are always changing, and they utilize many pathways to resist apoptosis and cell differentiation, which is an important reason for tumor deterioration, drug resistance, and recurrence. Therefore, actively exploring new mechanisms of anti-tumor drugs and developing efficient and low-toxicity therapeutics or adjuvant therapeutic drugs are important steps in the search for new anti-tumor solutions. Natural products are an important source of new drugs and actively exploring the possibilities of natural medicines to treat diseases is just as important as exploring emerging cancer treatment products.

### Modern science and technology promote the growth of natural medicines

Since the modern scientific and technological revolution, the development of active ingredients from natural products has increased due to the rapid development of genetic, cell, and fermentation engineering, and drug activities derived from natural products have been continuously explored. Obtaining active ingredients from natural products and developing them into clinical drugs is useful to fully promote the utilization of natural resources.

The potency and content of active ingredients in natural medicines are usually related to their place of origin. Most natural bioactive products face the problem of large-scale production to meet production demand, which constitutes one of the major obstacles for those promising drug candidates to eventually reach the clinic. Natural products with therapeutic activity should have a stable source to meet the marketing demand. For example, HHT is found in plants at extremely low levels, which is a problem that needs to be solved, and only China has achieved profitable production of HHT thus far [43, 44]. To introduce HHT to the world, an alternative source is needed to meet the growing demand. One of the 213 fungal strains isolated from *Cephalotaxus hainanensis* Li. has the ability to biosynthesize HHT and is expected to provide a sustainable source of HHT [110]. In addition to direct access, chemical synthesis is also a major route for the mass production of natural products. For example, the alkaloidal active ingredient camptothecin, which is derived from *Camptotheca acuminata* Decne, can either be isolated from the bark of *Camptotheca acuminata* Decne or synthesized by chemical methods [111]. Although natural products are known to be biologically active, their derivatives can also be actively explored for their biological activities, such as DIOXOL and HIMOXOL, OA derivatives, and etoposide, which is a derivative of podophyllin. In the framework of natural products, changing the chemical groups or chirality may improve therapeutic activity, which is one method of new drug development.

For natural drugs that have been identified to have therapeutic activity but poor oral bioavailability, low solubility, and weak membrane permeability, a number of new delivery technologies can be utilized, including liposomes, solid dispersions, nicotinic aldehyde gels, and nanoliposomes. For example, the oral bioavailability and anti-leukemia activity of Tan IIA can be substantially enhanced using biotinylated lipid bilayer-coated mesoporous silica nanoparticles (Bio-LB-MSNs) as a carrier [112]. Currently, many natural products for the treatment of APL are only at the stage of in vitro study and animal experiments, and they have not yet undergone clinical exploration and translation. Clinical data act as valid evidence for the promotion of natural products. Numerous clinical observations have proven that combined Chinese and Western medicine has value in clinical practice. For example, natural medicines supplemented with chemotherapy can improve the prognosis of patients and enhance their immunity, thus improving the quality of their survival and reducing their medical expenses to a certain extent. Under the premise of safety and effectiveness, natural medicine-related clinical trials should be vigorously promoted for the benefit of a wider group of patients.

### Intellectual property protection and promotion of natural medicine

Traditional medicines from China, India, Thailand, and many other countries play important roles in the treatment of diseases. These traditional medicines are not only used to treat diseases, but also they are part of the traditional culture of these countries. The collection of TCMs and traditional prescriptions, the collation and verification of evidence on their use, and the promotion of their application are important for combining Chinese and Western medicines. The Chinese medical community has been tirelessly exploring new medicines in TCM and seeking new applications for existing medicines, instead of completely disregarding traditional remedies or categorizing herbs as food additives. The advancement in our understanding of diseases not only allows for innovation in Western medicine therapy but also creates new possibilities for the “new application of ancient remedies”. Injectable ATO is one of the important achievements of the prescription collection of Chinese tradition. At present, there are still many unknown prescriptions in Chinese folk medicine, and many traditional medicines, including TCMs, have not yet entered the field of intellectual property protection for geographical and linguistic reasons. Traditional prescription provides one source of innovation. Collecting and organizing traditional prescriptions and explaining their mechanisms of action with theories of modern science can recognize the action pathways of natural medicines from different perspectives, and improve the vitality of medical innovation.

The United Nations Convention on Biological Diversity treaty has included the protection of traditional medicinal knowledge within its framework, and the efforts of international organizations have led some developed countries to pay attention to the protection of traditional medicinal knowledge and to implement specific rules with the help of existing intellectual property rights protection. At present, there is no law specifically protecting traditional medicine knowledge in China, and the protection methods are scattered. None of the existing intellectual property laws can systematically locate and comprehensively protect traditional medicine knowledge. Moreover, due to the scattered provisions, the protection effect is relatively weak and the scope of protection is narrow [113]. Developers from economically developed countries and regions rely on their scientific and technological knowledge to develop, utilize, improve, and innovate traditional medicine without permission. They use the evidence acquired from these investigations to develop new medicines and obtain intellectual property rights, mainly patents, as well as to obtain high commercial profits, resulting in the frequent loss of intellectual property rights of TCM, which is already faced with a lack of protection [114]. As a successful country in protecting traditional medicines, India has gradually established a unique intellectual property system to regulate the traditional medicine industry, while resisting biopiracy [115]. This is a good example that the development of natural products should be based on the protection of origin and intellectual property. The development of natural medicine should conducted on the premise of mutual benefit, and ultimately benefit for the world.

## Data Availability

The datasets presented in this review can be found in online repositories.

## Abbreviations

WHO: World Health Organization
APL: Acute promyelocytic leukemia
AML: Acute myeloid leukemia
PML: Promyelocytic leukemia
RARα: Retinoic acid receptor-α
CR: Complete remission
ATRA: All-trans retinoic
ATO: Arsenic trioxide
TCM: Traditional Chinese medicine
RIF: Realgar-indigo naturalis formula
HHT: Homoharringtonine
DFS: Disease-free survival
Ara-C: CytosineArabinoside
DNR: Daunorubicin
IDA: Idarubicin
RAS: Retinoic acid syndrome
HKL: Honokiol
MAG: Magnolol
ROS: Reactive oxygen species
DL: Dehydrocostus lactone
ART: Artesunate
ALT: Alanine transaminase
AST: Aspartate transaminase
BUN: Blood urea nitrogen
CREA: Creatinine concentrations
Tan IIA: Tanshinone IIA
OA: Oleanolic acid
UA: Ursolic acid
NF-κB: Nuclear factor kappa-B
CRO: Crocin
CRT: Crocetin
EGCG: Epigallocatechin gallate
ECG: Epicatechin gallate
EGC: Epigallocatechin
EC: Epicatechin
CMEMM: Crude methanolic extract of *Mucuna macrocarpa*
